# Cervical ependymoma en bloc resection

**DOI:** 10.3171/2023.6.FOCVID2390

**Published:** 2023-10-01

**Authors:** Matheus A. Bannach, Mateus N. F. Fernandes, Rodrigo Cavalcante

**Affiliations:** 1 Department of Surgery, Division of Neurosurgery, Medical School, Federal University of Goiás; and; 2 Medical School, Federal University of Goiás, Goiânia, Brazil

**Keywords:** ependymoma, microsurgery, surgical technique

## Abstract

A 58-year-old male was admitted to the authors’ department due to cervicothoracic pain and disequilibrium. Physical examination evidenced sensory and motor deficits in the lower limbs. MRI evidenced an expansive intramedullary lesion compatible with ependymoma. The nuances of this surgical access and the management of intradural tumors are discussed.

## Transcript

### 0:21 Ependymoma.

In this video we present a case of cervical ependymoma en bloc resection. The spinal ependymomas are WHO grade II tumors originated from the ependymal cells, representing 60% of all glial intramedullary tumors in adults. The cervical spine is the most common location for this tumor.^1–3^

### 0:41 Treatment.

The preferred treatment is surgical resection, total resection being achieved in approximately 50% of cases, with a high 5-year survival rate and rare recurrence following surgery.^3^

### 0:52 History and Presentation.

Our patient is a 58-year-old male, with long-standing cervicothoracic pain, worsening in the last 5 months, associated with imbalance and gait disturbance. On physical examination, he presented with tetraparesis grade 4 from the Medical Research Council scale;^4^ sensory dissociation, characterized by reduction of pain and temperature sensation; and preserved proprioception and fine touch. Associated with incoordination and gait instability. Positive Romberg sign, hyperreflexia, and Babinski sign were also seen.

In the MRI we can see a large expansive lesion from C4 to D1, with a hypointense sign on the inferior aspect of the tumor, characterizing the classic cap sign of the ependymoma. Syringomyelia is also seen in the thoracic spine.

### 1:45 Positioning and Patient Preparation.

The surgical planning involved a prone position with general anesthesia and head fixation using a Mayfield clamp. A combination of somatosensory and motor evoked potential was used for intraoperative neurophysiological monitoring. Midline incision with subperiosteal dissection of the muscle planes was performed, exposing the posterior aspects of the cervical vertebrae, followed by laminectomy from C4 to D1.

### 2:12 Dural Opening and Myelotomy.

The dura mater was opened in the midline, exposing the entire posterior aspect of the spinal cord, and fixated laterally with sutures. We see an enlargement of the spinal cord in the region of the tumor. Identifying the midline is a pivotal step on the surgery, which can be made through three mechanisms. The first is the anatomy of the vessels, with direct visualization of the small veins bilaterally coming from surface into the posterior median sulcus. The second way would be measuring half the distance between the two dorsal root entry zones laterally.^5^ The final way would be stimulating the posterior cord and locating the site of inversion of the action potential, demonstrating the midline. A large vein was positioned exactly above the sulcus, making it difficult to start the midline myelotomy, which was done at a superior level where the vein was displaced laterally. It is important trying to preserve this vein to avoid vascular complications postoperatively, since the normal tissue is already compressed.

Now we see midline coagulation to start incision. With a sharp scalpel we perform the myelotomy. Then we used blunt dissection to start developing a plane between tumor and normal spine. We already see a dark region indicating the initial aspect of the tumor. Here we see the placement of pia mater suture, in order to keep the myelotomy open, facilitating dissection of the tumor from the normal spine, while avoiding unnecessary traction. This helps the surgeon to keep the plane of dissection, and it is especially important in ependymomas, once it is a tumor that usually has a well-developed plane of dissection. But the main complications are associated with traction and lesions of the vascular nutrition of the normal spine.

### 4:10 Tumor Dissection.

On the central part of the tumor, we see much more adhesion than in the poles, indicating that this is probably the source of the tumor and limiting our ability to progress with the dissection without damaging normal tissue. Thus, we decided to perform another midline myelotomy on the inferior aspect of the tumor, locating the inferior limit and then connecting the two incisions in order to maintain the plane of dissection, avoiding entering the normal spine. Special attention is dedicated to the anterior aspect of the tumor, due to proximity of multiple motor structures. This gentle dissection helps to avoid complications and permanent deficits. The neurophysiological monitoring may show an acute drop on the evoked potentials; however, this should not be taken as definite lesion, neither should be used to indicate interruption of the procedure.^5^ Warm saline or papaverine pads can be placed on the spine, while the surgeon migrates to another site of the tumor. Most of the time the evoked potentials are restored after a few minutes. The D-wave is more relevant to assess potential lesion; however, in tumors with clear plane of cleavage, even if there is a major drop of signal, motor worsening tends to be transitory. We notice that the maneuver is to push the tumor away from the spine, and not the spine away from the tumor. This helps maintain the spinal cord in its place, avoiding traction and also lesions. The final adhesions of the tumor are released, completely surrounding the tumor and maintaining the pseudocapsule. This helps to ensure total resection and reduce the chance of recurrence. It might not be possible if there is tumor invasion, characteristic of a type 3 ependymoma. In these cases, debulking might be advisable.

Now we see the final detachment of the tumor from the spinal cord and its removal from the spinal cord cavity. We see no bleeding in the tumor cavity; therefore, there is no need for any coagulation, avoiding additional deficits. Finally, the dura mater was closed with continuous suture and vertebrae fixation was performed using lateral mass screws from C4 to C6 and pedicle screws on D1 vertebrae. Autograft was used for arthrodesis.

Although laminoplasty has been advocated in recent years, this case involved four laminectomies in a spinal junctional segment; therefore, we preferred fusion to diminish the risk of kyphosis. Fusion should be considered in cases with more than three laminectomies, spine deformity preoperatively, facetectomy greater than 50%, C2 laminectomy, and spinal junction surgeries.^6^

### 7:04 Postoperative Outcomes and Images.

Pathology confirmed a type 2 ependymoma. Postoperatively, the patient presented with improvement of balance, while maintaining strength and had resolution of cervical thoracic pain with no major deficits. In the 6-month follow-up, he had improvement of gait, but maintained sensory deficits.

Postoperative MRI showed complete resection of the tumor and partial resolution of syringomyelia. This is the patient’s improvement of gait, maintaining functionality and no additional deficits, only minor instability of gait.

## Acknowledgments

We thank the staff of the Neurosurgery Laboratory at Faculdade de Medicina–Universidade Federal de Goiás for assistance with transcript and video preparation.

## Disclosures

The authors report no conflict of interest concerning the materials or methods used in this study or the findings specified in this publication.

## Author Contributions

Primary surgeon: Cavalcante. Assistant surgeon: Bannach. Editing and drafting the video and abstract: Bannach, Fernandes. Critically revising the work: all authors. Reviewed submitted version of the work: Bannach, Fernandes. Supervision: Cavalcante, Bannach.

## Supplemental Information

### Patient Informed Consent

The necessary patient informed consent was obtained in this study.
